# Supplementary material for: Preparation of pod-shaped TiO2 and Ag@TiO2 nano burst tubes and their photocatalytic activity
Source: R Soc Open Sci. 2019 Sep 4;6(9):191019. doi: 10.1098/rsos.191019 (PMC6774943; doi:10.1098/rsos.191019)
Supplement: Supplementary material [file rsos191019supp1.docx]

**Preparation of pod-shaped TiO_2_ and Ag@TiO_2_ nano burst tubes and their photocatalytic activity**

Shang Wang‡^1^, Zhaolian Han‡^1^, Tingting Di^2^, Rui Li^1^, Siyuan Liu^1^ and Zhiqiang Cheng*^1^

^1.^College of Resources and Environment, Jilin Agricultural University, Changchun 130118, People’s Republic of China.

^2.^Northeast Electric Power Design Institue Co.,LTD. of China Power Engineering Consulting Group, Changchun 130021, People’s Republic of China.

*Correspondence to: Zhiqiang Cheng (czq5974@163.com)

‡Shang Wang and Zhaolian Han contribute to the work equally.


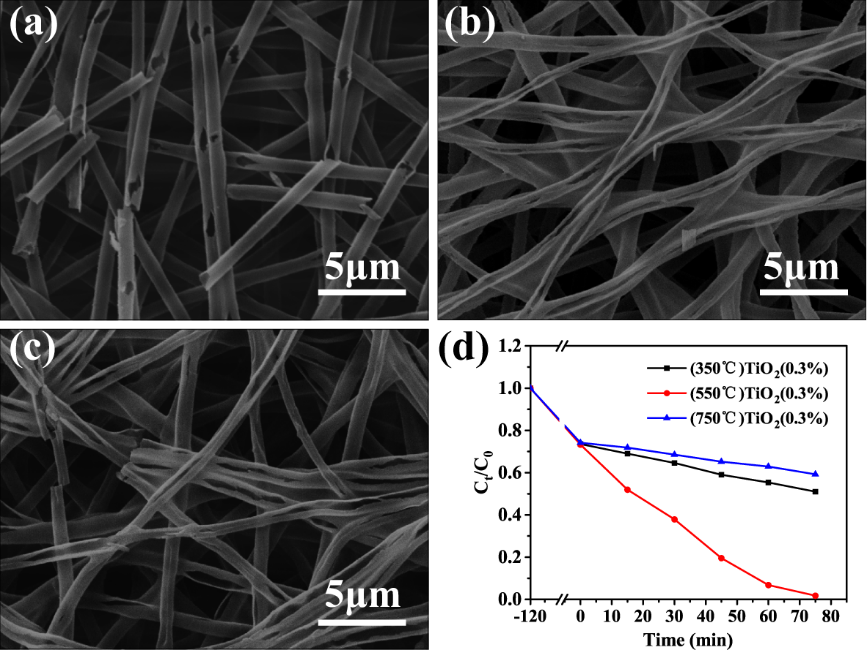
Fig. S1 SEM image of calcination temperature of 350 °C (a), 550 °C (b), 750 °C (c) and photocatalytic activity of photocatalyst for photodegradation of RhB under ultraviolet light irradiation (d).

When the calcination temperature is 350 °C, Fig. S1(a) shows that only a small portion of the TiO_2_ nanotubes is cracked. The reason is that H_2_O and CO_2_ produced by the decomposition of H_2_C_2_O_4_ and PS are very slow at low temperature calcination. Therefore, the gas gently escapes from the surface of the TiO_2_ NBTs, resulting in TiO_2_ not being well cracked. However, when the temperature rises to 550 °C (Fig. S1(b)) and 750 °C (Fig. S1(c)), H_2_C_2_O_4_ and PS will rapidly decompose the H_2_O and CO_2_ gases generated. The impact of a large amount of gas will cause the surface of the TiO_2_ nanotube to crack and form a pod-shaped TiO_2_ NBTs. Therefore, there is almost no difference in the SEM pattern formed by calcination at 550 °C and 750 °C. The catalytic degradation experiments were carried out on three samples. The experimental results in Fig. S1(d) show that the degradation efficiency of the TiO_2_ NBTs calcined at 550 °C is 94.0%, while the degradation efficiency at 550 °C and 750 °C is 30.7. % and 20.1%.


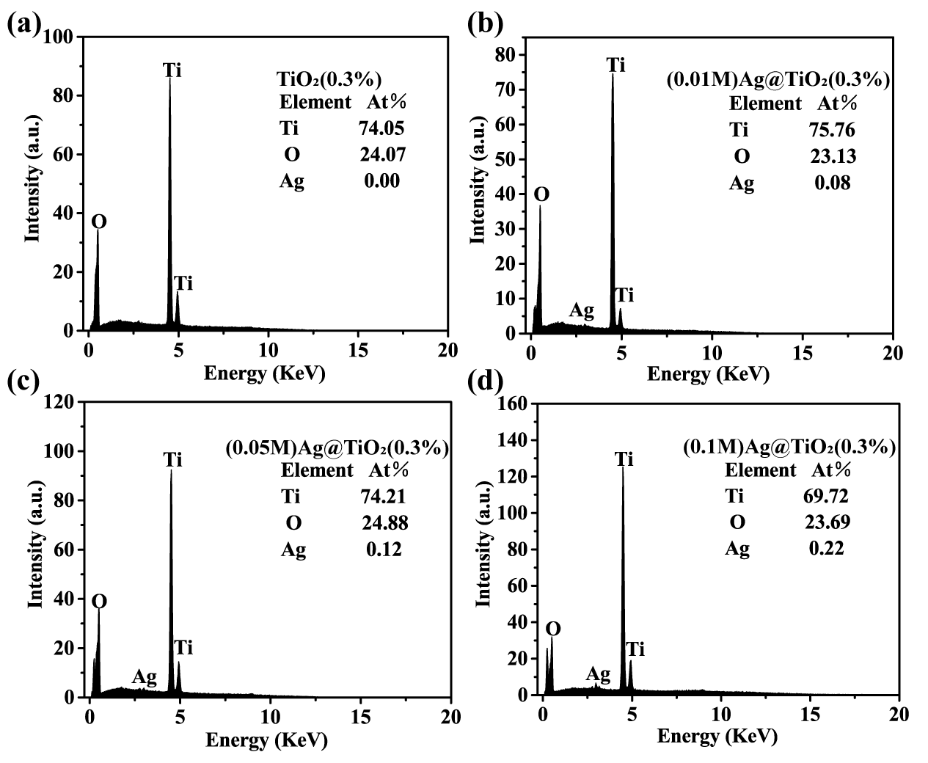
Fig. S2 The EDX spectra of (a) TiO_2_(0.3%),(b) (0.01M)Ag@TiO_2_(0.3%), (c) (0.05M)Ag@TiO_2_(0.3%) and (d) (0.10M)Ag@TiO_2_(0.3%).

From the Fig. S2 we can observe the EDX spectrum of the TiO_2_ with different concentration of AgNO_3_. The peaks of the Ti, O and Ag are clearly observable and the atom percent was 74:24:0; 76:23:0.08; 74:25:0.12 and 70:24:0.22, respectively. Result of increasing concentration of AgNO_3_, the amount of deposited AgNPs on the surface of the Ag@TiO_2_ NBTs increased. This due to the higher concentration of AgNO_3_ solution causes more AgNPs to accumulate on the surface of the TiO_2_ NBTs, which leads to an increase in particle size, resulting in a decrease in the specific surface area of the particles and the surface active sites. Therefore, when the atomic percentage of the peaks of Ti, O and Ag is 74:25:0.12, the sample has the strongest catalytic ability.


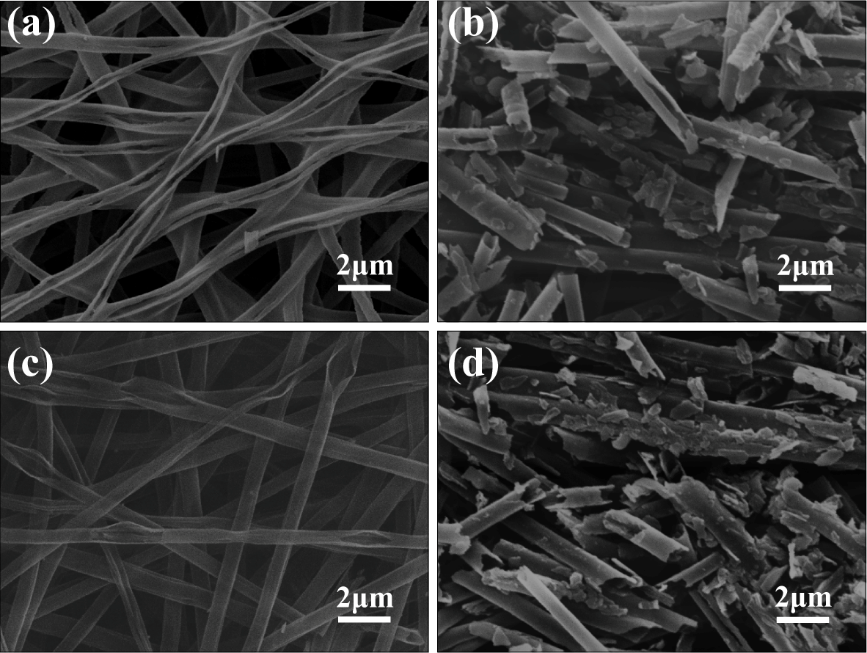
Fig. S3 SEM image of pod-shaped TiO_2_(0.3%)(a-b) and (0.05M)Ag@TiO_2_ (0.3%)(c-d) NBTs before and after the catalytic experiment.

As shown in the SEM image of Fig. S3, the morphology of the pod-shaped TiO_2_(0.3%) NBTs (a) and (0.05M)Ag@TiO_2_(0.3%) NBTs (c) was clear before the catalytic experiment. The nanotubes is evenly connected. However, when the reaction was completed, the recovered pod-shaped TiO_2_(0.3%) NBTs (b) and (0.05M)Ag@TiO_2_(0.3%) NBTs (d) were subjected to scanning electron microscopy. The results showed that the structure of the catalyst after the reaction changed. The sample is transformed from a uniformly cracked nanoburst into a broken nanoburst. Therefore, the morphology of the nano burst tubes of pod-shaped TiO_2_(0.3%) changes with the degradation experiment.
